# Supplementary material for: The Small RNA Universe of Capitella teleta
Source: Front Mol Biosci. 2022 Feb 25;9:802814. doi: 10.3389/fmolb.2022.802814 (PMC8915122; doi:10.3389/fmolb.2022.802814)
Supplement: Supplementary file 1 [file DataSheet1.ZIP › Supplement/known/CAPTEscaffold_664_26671.pdf]

Provisional ID	:	CAPTEscaffold_664_26671
Score total	:	5.4
Score for star read(s)	:	-1.3
Score for read counts	:	0
Score for mfe	:	2.1
Score for randfold	:	1.6
Score for cons. seed	:	3
Total read count	:	5121
Mature read count	:	4488
Loop read count	:	0
Star read count	:	633

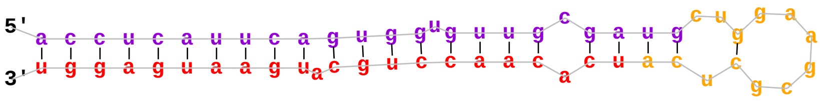

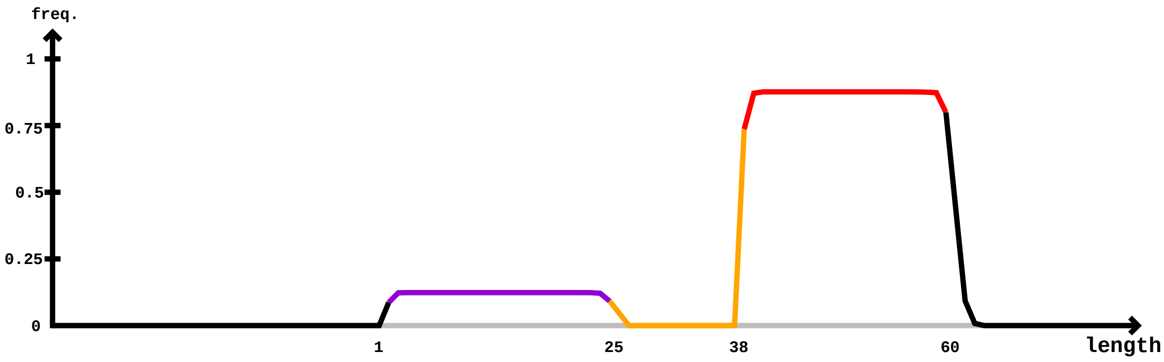


```
                                              Star                            Mature
5'- gcucauuucuuuggcaaucugcccagcgaucuuaccucauucagugguguugcgaugcuggaagcgcucaucacaaccugcaugaaugagguaaggucauggagcaugcgc  -3'   obs
    gcucauuucuuuggcaaucugcccagcgaucuuaccucauucagugguguugcgaugcuggaagcgcucaucacaaccugcaugaaugagguaaggucauggagcaugcgc         exp
    gcucauuucuuuggcaaucugcccagcgaucuuaccucauucagugguguugcgaugcuggaagcgcucaucacaaccugcaugaaugagguaaggucauggagcaugcgc         known
    .............(((...((((((..((((((((((((((((((((.((((.((((..(......).)))).)))))))).))))))))))))))))).))).))))))..   reads    mm       sample

    .................................accucauucagugguguugcg.........................................................   1        0        seq
    .................................accucauucagugguguugcga........................................................   11       0        seq
    .................................accucauucagugguguugcgG........................................................   1        1        seq
    .................................accucauucagugguguugcgau.......................................................   38       0        seq
    .................................Cccucauucagugguguugcgaug......................................................   1        1        seq
    .................................Nccucauucagugguguugcgaug......................................................   1        1        seq
    .................................Gccucauucagugguguugcgaug......................................................   3        1        seq
    .................................accucauucagugguguugcAaug......................................................   1        1        seq
    .................................accucauAcagugguguugcgaug......................................................   1        1        seq
    .................................accucauucagugguguugcgaug......................................................   380      0        seq
    .................................accucauucagugguguugcgauU......................................................   5        1        seq
    .................................accucauucagugguguugcgaugU.....................................................   2        1        seq
    .................................accucauucagugguguugcgaugA.....................................................   1        1        seq
    ..................................ccucauucagugguguugcgau.......................................................   111      0        seq
    ..................................ccuUauucagugguguugcgau.......................................................   1        1        seq
    ..................................Ncucauucagugguguugcgau.......................................................   1        1        seq
    ..................................ccucauucagugguguugcgaug......................................................   64       0        seq
    ..................................ccucauucagugguguugcgauU......................................................   5        1        seq
    ..................................ccucauucagugguguugcgaugU.....................................................   2        1        seq
    ...................................cucauucagugguguugcgauU......................................................   2        1        seq
    ...................................cucauucagugguguugcgaug......................................................   1        0        seq
    ......................................................................ucacaaccugcaugaaug.......................   2        0        seq
    ......................................................................ucacaaccugcaugaauga......................   4        0        seq
    ......................................................................ucacaaccugcaugaaugag.....................   10       0        seq
    ......................................................................ucGcaaccugcaugaaugagg....................   2        1        seq
    ......................................................................ucacaaccugcaugaaugagg....................   348      0        seq
    ......................................................................ucacaaccugcauAaaugagg....................   1        1        seq
    ......................................................................uAacaaccugcaugaaugagg....................   1        1        seq
    ......................................................................Acacaaccugcaugaaugagg....................   1        1        seq
    ......................................................................ucacaaccAgcaugaaugagg....................   1        1        seq
    ......................................................................Gcacaaccugcaugaaugagg....................   1        1        seq
```

Star Mature

```
gcucauuucuuuggcaaucugcccagcgaucuuaccucauucagugguguugcgaugcuggaagcgcucaucacaaccugcaugaaugagguaaggucauggagcaugcgc
......................................................................ucacaaccugcaugaauAagg....................    1     1    seq
......................................................................ucacaaccuCcaugaaugaggu...................    2     1    seq
......................................................................Acacaaccugcaugaaugaggu...................    18    1    seq
......................................................................ucacaaccugcaugaaugaggA...................    8     1    seq
......................................................................ucGcaaccugcaugaaugaggu...................    23    1    seq
......................................................................ucaAaaccugcaugaaugaggu...................    1     1    seq
......................................................................ucacaaccCgcaugaaugaggu...................    1     1    seq
......................................................................ucacaaGcugcaugaaugaggu...................    1     1    seq
......................................................................uUacaaccugcaugaaugaggu...................    2     1    seq
......................................................................ucacaacUugcaugaaugaggu...................    3     1    seq
......................................................................ucacaaccugcaugaauCaggu...................    1     1    seq
......................................................................ucacaaccugcaugaaAgaggu...................    3     1    seq
......................................................................ucacaGccugcaugaaugaggu...................    1     1    seq
......................................................................ucacaaccugcaugaaugagCu...................    1     1    seq
......................................................................ucacaaccugcauUaaugaggu...................    1     1    seq
......................................................................ucacaaccugcaugaaugaAgu...................    1     1    seq
......................................................................ucacaaccugcGugaaugaggu...................    1     1    seq
......................................................................ucacaaccugcaugaUugaggu...................    1     1    seq
......................................................................ucacaaccugcaugaaugaggG...................    2     1    seq
......................................................................ucacaaccugUaugaaugaggu...................    3     1    seq
......................................................................ucacaaccugcauAaaugaggu...................    7     1    seq
......................................................................ucacaaccugcaugaaugaUgu...................    1     1    seq
......................................................................ucacaaccugAaugaaugaggu...................    1     1    seq
......................................................................ucacaaccugcaCgaaugaggu...................    2     1    seq
......................................................................uAacaaccugcaugaaugaggu...................    1     1    seq
......................................................................ucacaaccugcaugUaugaggu...................    1     1    seq
......................................................................Ncacaaccugcaugaaugaggu...................    1     1    seq
......................................................................ucacaaccugcaugaauAaggu...................    3     1    seq
......................................................................ucacaaccugcaugaaugaggu...................    2725  0    seq
......................................................................ucacaaccugcaugaaCgaggu...................    2     1    seq
......................................................................ucacaaccugcauAaaugaggua..................    1     1    seq
......................................................................ucacaaccugcaugaaugagguU..................    14    1    seq
......................................................................ucacaaccugcaugaaugaAgua..................    1     1    seq
......................................................................Gcacaaccugcaugaaugaggua..................    1     1    seq
......................................................................ucacaaccugcaugaauAaggua..................    1     1    seq
......................................................................ucGcaaccugcaugaaugaggua..................    1     1    seq
......................................................................Acacaaccugcaugaaugaggua..................    2     1    seq
......................................................................ucacaaccugcaugaaugaggua..................    235   0    seq
......................................................................ucacaaccugcaugaauCaggua..................    1     1    seq
......................................................................Gcacaaccugcaugaaugagguaa.................    1     1    seq
......................................................................ucacaaccugcaugaaugagguaa.................    236   0    seq
......................................................................ucacaaccugcaugaaugagguaU.................    52    1    seq
......................................................................ucGcaaccugcaugaaugagguaa.................    1     1    seq
......................................................................ucacaaccugUaugaaugagguaa.................    2     1    seq
......................................................................ucaGaaccugcaugaaugagguaa.................    1     1    seq
......................................................................ucacaaccugcauAaaugagguaa.................    1     1    seq
......................................................................Acacaaccugcaugaaugagguaa.................    1     1    seq
......................................................................ucacaaccugcaugaaugagguGa.................    1     1    seq
......................................................................ucacaaccugcaugaaugagguaC.................    2     1    seq
......................................................................ucacaaccugcaugaaugagguaaU................    9     1    seq
......................................................................ucacaaccugcaugaaugagguaaA................    17    1    seq
.......................................................................cacaaccugcaugaaugagg....................    23    0    seq
.......................................................................cacaaccugcaugaaugaggu...................    321   0    seq
.......................................................................cacaaccugcauAaaugaggu...................    1     1    seq
.......................................................................cacaaccugcaugaUugaggu...................    1     1    seq
.......................................................................Uacaaccugcaugaaugaggu...................    1     1    seq
.......................................................................cacaaccugcaugaaCgaggu...................    2     1    seq
.......................................................................cacaaccugcUugaaugaggu...................    1     1    seq
.......................................................................cacaaccugcaugaaAgaggu...................    1     1    seq
.......................................................................cacaaAcugcaugaaugaggu...................    1     1    seq
.......................................................................cacaaccugcaugaaugaggua..................    200   0    seq
.......................................................................cacGaccugcaugaaugaggua..................    1     1    seq
.......................................................................cacaaccugcaugaauAaggua..................    1     1    seq
.......................................................................cacaaccugcaugaaugagguU..................    4     1    seq
.......................................................................cacaaccugcGugaaugaggua..................    1     1    seq
.......................................................................cacaaccugcaugaaugagguaU.................    12    1    seq
.......................................................................cacaaccugcauAaaugagguaa.................    1     1    seq
.......................................................................cacaaccugcaugaaugagguaa.................    109   0    seq
.......................................................................cacaaccugcaugaaugagguaaA................    3     1    seq
.......................................................................cacaaccugcaugaaugagguaag................    1     0    seq
```

```
                                  Star                                 Mature
gcucauuucuuuggcaaucugcccagcgaucuuaccucauucagugguguugcgaugcuggaagcgcucaucacaaccugcaugaaugagguaaggucauggagcaugcgc
.......................................................................cacaaccugcaugaaugagguaaU................    6    1    seq
........................................................................acaaccugcaugaaugaggu...................    4    0    seq
........................................................................acaaccugcaugaaugaggua..................    3    0    seq
........................................................................acaaccugcaugaaugagguaa.................   14    0    seq
........................................................................acaaccugcaugaaugagguaaA................    3    1    seq
........................................................................acaaccugcaugaaugagguaaU................    1    1    seq
........................................................................acaaccugcaugaaugagguaag................    1    0    seq
```
